# High frequency of additional gene mutations in acute myeloid leukemia with *MLL* partial tandem duplication: *DNMT3A* mutation is associated with poor prognosis

**DOI:** 10.18632/oncotarget.5202

**Published:** 2015-09-08

**Authors:** Hsiao-Wen Kao, Der-Cherng Liang, Ming-Chung Kuo, Jin-Hou Wu, Po Dunn, Po-Nan Wang, Tung-Liang Lin, Yu-Shu Shih, Sung-Tzu Liang, Tung-Huei Lin, Chen-Yu Lai, Chun-Hui Lin, Lee-Yung Shih

**Affiliations:** ^1^ Division of Hematology-Oncology, Department of Internal Medicine, Chang Gung Memorial Hospital at Linkou, Taoyuan, Taiwan; ^2^ Graduate Institute of Clinical Medical Sciences, College of Medicine, Chang Gung University, Taoyuan, Taiwan; ^3^ Division of Pediatric Hematology-Oncology, Mackay Memorial Hospital, Taipei, Taiwan; ^4^ College of Medicine, Chang Gung University, Taoyuan, Taiwan

**Keywords:** *MLL*-PTD, acute myeloid leukemia, gene mutation, *DNMT3A* mutation

## Abstract

The mutational profiles of acute myeloid leukemia (AML) with partial tandem duplication of *mixed-lineage leukemia* gene (*MLL*-PTD) have not been comprehensively studied. We studied 19 gene mutations for 98 patients with *MLL*-PTD AML to determine the mutation frequency and clinical correlations. *MLL*-PTD was screened by reverse-transcriptase PCR and confirmed by real-time quantitative PCR. The mutational analyses were performed with PCR-based assays followed by direct sequencing. Gene mutations of signaling pathways occurred in 63.3% of patients, with *FLT3*-ITD (44.9%) and *FLT3*-TKD (13.3%) being the most frequent. 66% of patients had gene mutations involving epigenetic regulation, and *DNMT3A* (32.7%), *IDH2* (18.4%), *TET2* (18.4%), and *IDH1* (10.2%) mutations were most common. Genes of transcription pathways and tumor suppressors accounted for 23.5% and 10.2% of patients. *RUNX1* mutation occurred in 23.5% of patients, while none had *NPM1* or double *CEBPA* mutation. 90.8% of *MLL*-PTD AML patients had at least one additional gene mutation. Of 55 *MLL*-PTD AML patients who received standard chemotherapy, age older than 50 years and *DNMT3A* mutation were associated with inferior outcome. In conclusion, gene mutations involving DNA methylation and activated signaling pathway were common co-existed gene mutations. *DNMT3A* mutation was a poor prognostic factor in *MLL*-PTD AML.

## INTRODUCTION

The *mixed-lineage leukemia* (*MLL*) gene, now officially named *lysine* (K)*-specific methyltransferase 2A (KMT2A)* gene, locates on chromosome 11q23, and encodes a protein of which the SET domain has histone methyltransferase activity that specifically methylates lysine 4 on histone H3, a modification typically associated with transcriptionally active regions of chromatin [1]. The MLL protein acts as a maintenance factor for the homeobox (HOX) group of proteins, which play an important role in specifying cell fate during development and hematopoiesis [2]. *MLL* gene was initially recognized as a recurrent locus of chromosomal translocation, having more than 85 distinct translocations with different fusion partners [3]. The *MLL* gene may also be rearranged to generate tandem duplications, the *MLL* partial tandem duplication (*MLL*-PTD), which is a cryptic gene rearrangement that most commonly duplicates exons 3 through 9 (e9e3) or exons 3 through 11 (e11e3) giving rise to an in-frame repetition and procured an elongated protein [4]. Like wild-type MLL, the MLL-PTD retained the transcriptional activity of histone H3 lysine 4 methyltransferase within the C-terminal SET domain and impact with the transcriptional regulation of *HOX* genes and other tumor suppressor genes [5, 6].

*MLL*-PTD occurred in about 5% to 7% of adult *de novo* AML, mostly in patients with normal karyotypes [7–10]. In several but not all studies, patients with *MLL*-PTD had shorter event-free survival (EFS) and overall survivals (OS) [7, 8, 11]. The mechanism of how *MLL*-PTD contributes to AML and the genetic basis of *MLL*-PTD associated AML is still unclear. In recent years, novel mutated genes involving epigenetic regulators have been described to occur recurrently in AML [12–14]. Although with its epigenetic regulator function, the cooperation of *MLL*-PTD with other epigenetic regulator genes such as *TET2, IDH1, IDH2, DNMT3A, ASXL1* and *EZH2* have not been comprehensively studied. Furthermore, the data on additional molecular alterations in *MLL*-PTD associated AML is scarce and thus their genetic background is largely unknown. We thus examined a wide spectrum of gene mutations of epigenetic regulators, tumor suppressor genes, signal transactivation (class I) and transcription pathways (class II) on bone marrow cells from patients with *de novo MLL*-PTD associated AML at the initial diagnosis. The status of gene mutations was correlated with the clinico-hematologic features and outcomes to determine their clinical relevance in patients with *MLL*-PTD associated AML.

## RESULTS

### Characterization of *MLL*-PTD AML patients

Median age of the 98 patients was 55 years (range 6–86). The ratio of male to female was 1:1. The clinical features of 98 *MLL*-PTD AML patients are listed in Table 1. The estimated median OS was 5.4 months. Fifty-five patients received standard AML protocol (daunomycin 60 mg/m^2^ for 3 days and cytarabine 100 mg/m^2^ for 7 days as induction therapy in adults or TPOG-AML97A for children with an age less than 18 years) [18]. Of the 55 patients who received standard AML protocol, 32 achieved complete hematologic remission. Eight patients received allogeneic stem cell transplantation (Supplementary Table S3). The median follow-up time for 55 patients who received standard chemotherapy was 11.0 months (range 0.5–114.2), and the estimated median EFS and OS were 5.2 months (95% CI 0–10.7) and 11.0 months (95% CI 8.6–13.3), respectively.

**Table 1:** Characteristics of 98 patients with *MLL*-PTD AML

Features	Results
**Median age (years)**	55.0 (6–86)
**Age groups**	
<18 years	5 (5.1%)
18–59 years	47 (48.0%)
>=60 years	46 (46.9%)
**Male/female (ratio)**	49/49 (1.0)
**Blood counts, median (range)**	
WBC count (×10^9^/l)	30.5 (0.8–451.4)
Hemoglobin level (g/l)	74.0 (33.0–137.0)
Platelet count (×10^9^/l)	46.0 (1.0–900.0)
Circulating blast (%)	65.7 (0–98.3)
Marrow blast (%)	79.4 (34.7–99.4)
**FAB subtype**	
M0	5 (5.1%)
M1	24 (24.5%)
M2	43 (43.9%)
M4	17 (17.3%)
M5	5 (5.1%)
M6	3 (3.1%)
M7	1 (1.0%)
**ELN cytogenetic risk groups**	
Favorable	0 (0%)
Intermediate 1	56 (73.6%)
Intermediate 2	19 (25.0%)
Adverse	1 (1.3%)
Not evaluated	22
**Number of cytogenetic alterations**	
0 (Normal karyotype)	56 (73.6%)
1	13 (17.1%)
2	6 (7.9%)
3 or more	1 (1.3%)
Not evaluated	22

FAB: French-American-British, ELN: European LeukemiaNet

### Cytogenetic alterations of *MLL-PTD* AML patients

Seventy-six patients had available cytogenetic data. Twenty of 76 (26.3%) patients had at least one chromosomal abnormality; 13 (17.1%) patients had one chromosome abnormality, and seven (9.2%) had two or more chromosomal abnormalities. The most frequent cytogenetic changes were trisomy 8 (*n* = 4) and trisomy 11 (*n* = 3). None of the patients were in the ELN favorable risk group. Fifty-six (73.6%) patients with normal karyotypes were categorized in the European LeukemiaNet (ELN) intermediate-1 group, 19 (25.0%) patients in the ELN intermediate-2 group, and one patient was in the ELN adverse risk group (1.3%; 46, XY, del(3)(p13p22), −4, +8 [12]).

### Concomitant gene mutations involving epigenetic regulators in *MLL*-PTD AML

**Figure 1:**
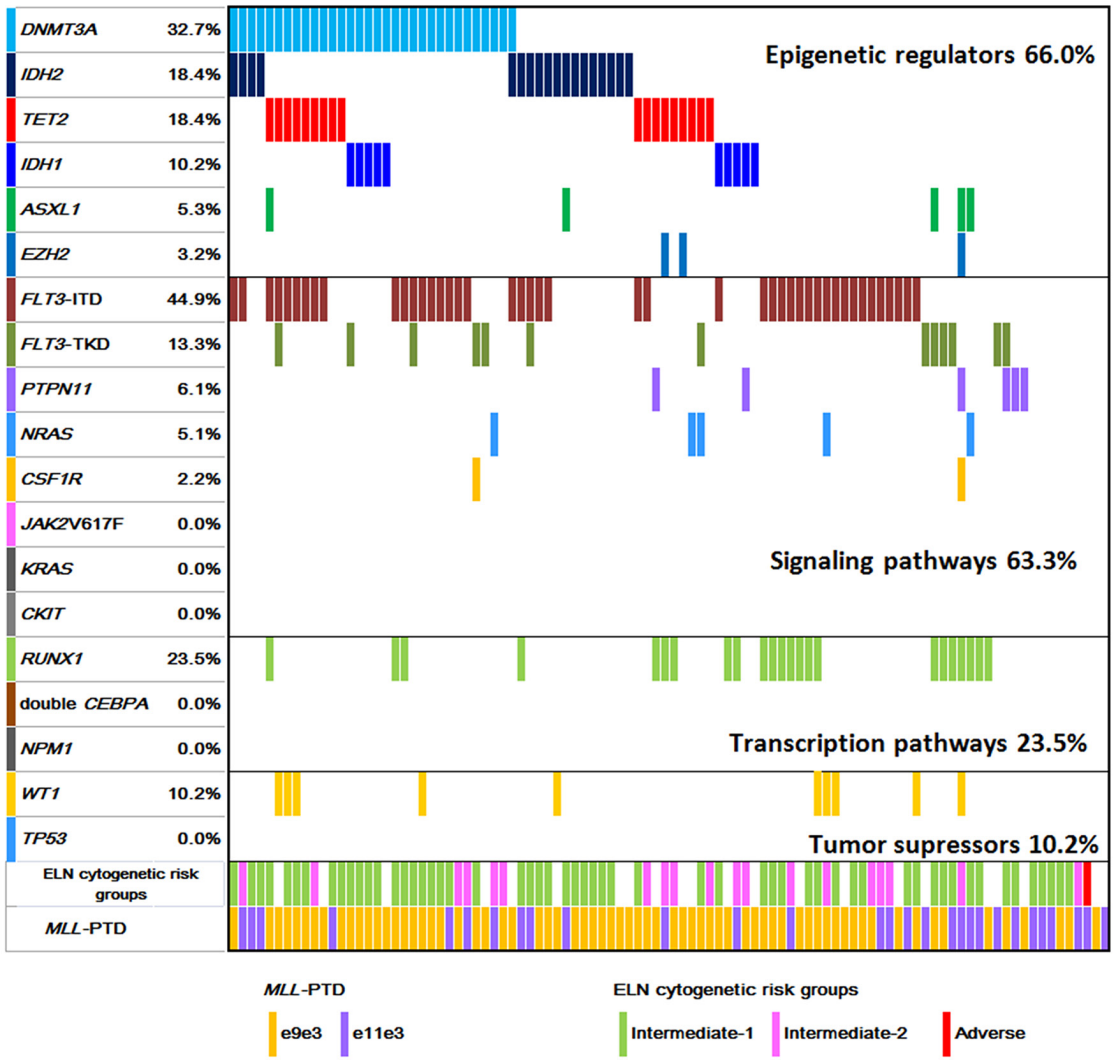
Diagram of gene mutations in patients with *MLL*-PTD AML The additional gene mutation status and cytogenetic risk groups in *MLL*-PTD AML patients at the initial diagnosis are illustrated.

The gene mutational status of *DNMT3A, TET2, IDH1, IDH2, ASXL1, EZH2,* and other gene mutations in *MLL*-PTD associated AML patients is shown in Figure 1.

*DNMT3A* mutations were found in 32 of 98 (32.7%) patients. Most *DNMT3A* mutations clustered in the methyltransferase domain at amino acid R882. There were 22 mutations (67.7%) at amino acid R882 in exon 23 (R882H, *n* = 15; R882C, *n* = 6; R882S, *n* = 1). Twenty-nine mutations were single nucleotide substitutions leading to missense (*n* = 28, 87.5%) and nonsense mutations (*n* = 1, 3.1%). Three patients had frameshift mutations resulted from insertions or deletions (*n* = 3, 9.4%).

*TET2* mutations were identified in 18 of 94 (18.4%) patients. Of the 18 mutated cases, three carried two *TET2* mutations. In total, 21 distinct mutations were detected, which were composed of nine point mutations (42.9%), eight deletions (38.1%), and four insertions (19.0%). These alterations led to 12 frame-shift mutations, two nonsense and seven missence mutations. These mutations spread across all the exons of *TET2*, with the majority of mutations (14/21, 66.7%) located in exons 3 and 8.

*IDH2* mutations were present in 18 of 98 (18.4%) patients, with *IDH2*-R140Q in 14 patients, and *IDH2*-R172 in four patients (3 R172K, 1 R172S). *IDH1* mutations occurred in 10 of 98 (10.2%) patients, with four R132H, three R132C, two R132S, and one R132L.

*ASXL1* and *EZH2* mutations were detected less frequently. Five of 94 (5.3%) patients had *ASXL1* mutations. Three patients carried protein-truncating (two frame-shifts and one nonsense) mutations and two patients had missence mutations. Three out of 94 (3.2%) patients had *EZH2* mutations with one each of L378Kfs*6, Y731C and C565Y.

Taken together, among the 94 patients examined for all the six genes of epigenetic regulators, 66.0% of patients had at least one gene mutation; 62.1% (59/95) involved in DNA methylation including *DNMT3A, TET2, IDH1*, and *IDH2* mutations. Mutations in genes of histone modifiers, *ASXL1* and *EZH2*, were rare (7/94, 7.4%) in *MLL*-PTD AML.

### Other concomitant gene mutations in *MLL*-PTD AML

*FLT3*-ITD and *FLT3*-TKD mutations were detected in 44.9% (44/98) and 13.3% (13/98) of patients, respectively. Other mutations of class I signaling pathways occurred less frequently, with the frequencies of 6.1% for *PTPN11*, 5.1% for *NRAS* and 2.2% for *CSF1R*. None had *JAK2*^V617F^, *KRAS* or *CKIT* mutations. For class II mutations of transcription pathway, *RUNX1* mutations were detected in 23.5% (23/98) patients, including 16 frame-shift mutations leading to premature stop codon, three missense mutations, and four nonsense mutations. Single *CEBPA* mutations were found in four patients but none had double *CEBPA* mutations. No *NPM1* mutation was detected. Among tumor suppressor genes, *WT1* mutations were detected in 10.2% (10/98), and none was positive for *TP53* mutation. Considering the functional classes of the genes, mutations in class I, class II and tumor suppressor genes occurred in 63.3%, 23.5% and 10.2% of patients, respectively. Together, 91.8% (90/98) of patients had at least one additional gene mutations examined in *MLL*-PTD AML patients.

### Correlations between gene mutations and clinical parameters

The clinical parameters including age, sex, hemoglobin level, platelet counts, white blood cell (WBC) counts, percentage of circulating blasts or marrow blasts, and cytogenetic risk groups were correlated with gene mutation status of *DNMT3A, TET2, IDH1/IDH2, RUNX1* and *FLT3*-ITD. *TET2* mutated patients were significantly older (median 71 vs. 54 years, *P* = 0.004), and the presence of mutations of *DNMT3A* (61 vs. 54 years, *P* = 0.053) had a trend of association with older age. *IDH1/IDH2* mutations were associated with a lower WBC counts (11.3 × 10^9^/L and 41.5 × 10^9^/L, *P* = 0.059). *MLL*-PTD AML patients with *FLT3*-ITD had significantly higher WBC counts (63.5 vs. 14.9 × 10^9^/L, *P* = 0.001), circulating blasts (71.8 vs. 61.7%, *P* = 0.036) and marrow blasts (82.9 vs. 69.3%, *P* = 0.001) than patients without *FLT3*-ITD. No significant differences were found with regard to other clinical characteristics according to the mutation status of each gene (Supplementary Table S4).

### Survival analysis

**Figure 2:**
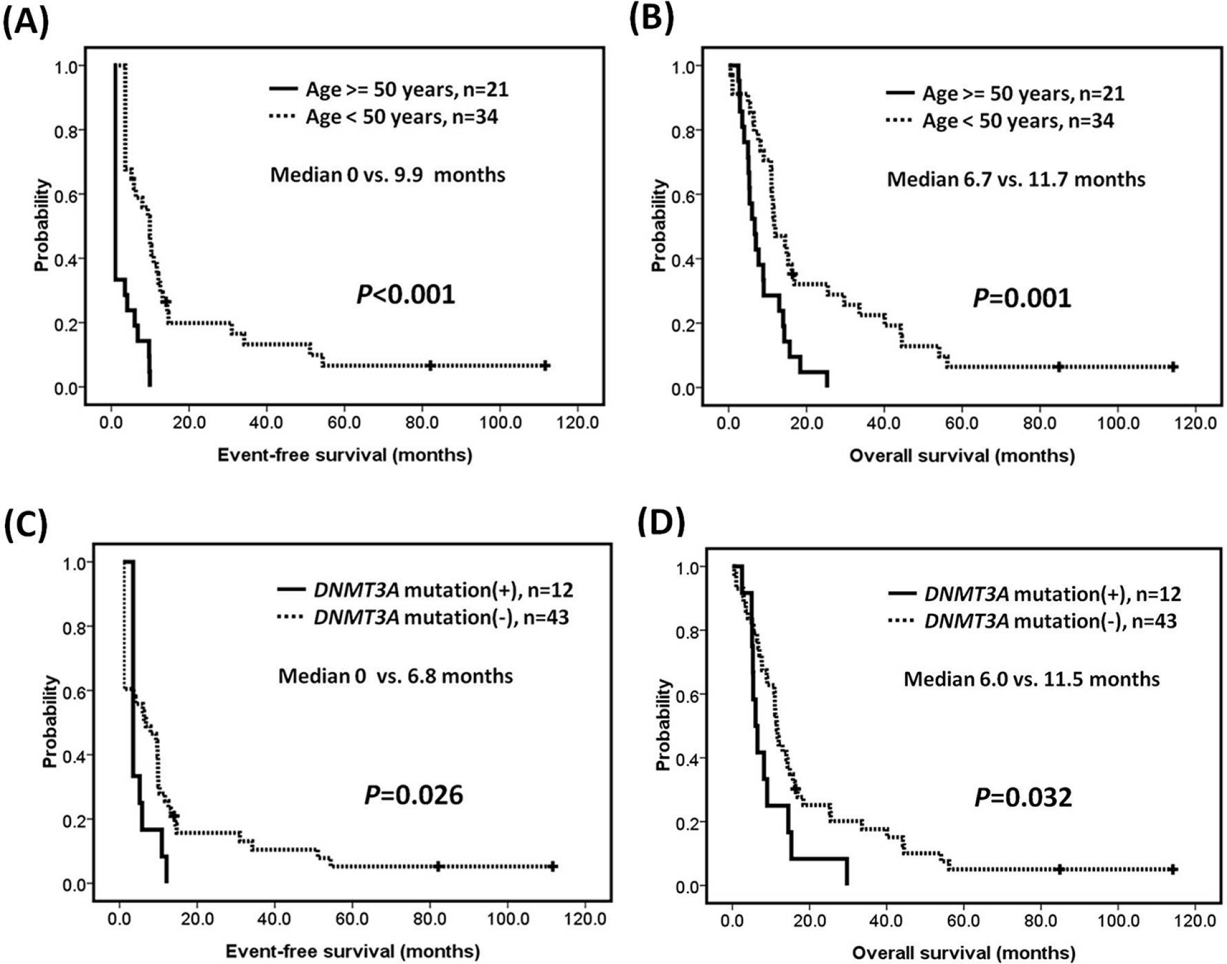
Survivals of patients with *MLL*-PTD AML Kaplan-Meier estimates of **A.** event-free survival and **B.** overall survival according to age, and **C.** event-free survival and **D.** overall survival according to the mutation status of *DNMT3A* in patients with *MLL*-PTD associated AML.

Outcome analyses were restricted to patients receiving standard therapy (55/98, 56.1%). We assessed the impact of age (>50 vs <= 50 years), *FLT3*-ITD, *IDH1/IDH2, RUNX1, DNMT3A*, and *TET2* mutations on EFS and OS. Patients more than 50 years old had significantly shorter EFS (median 0 vs. 9.9 months, *P* < 0.001) and OS (median 6.7 vs. 11.7 months, *P* = 0.001) (Figures 2A–2B). *MLL*-PTD AML patients with *DNMT3A* mutations had inferior EFS (median 0 vs. 6.8 months, *P* = 0.026) and OS (median 6.0 vs. 11.5 months, *P* = 0.032) compared to those without *DNMT3A* mutations (Figure 2C–2D). The mutational status of *FLT3*-ITD, *RUNX1, IDH1/IDH2, TET2*, or *ASXL1* did not have significant influence on EFS and OS (Supplementary Figure S1).

## DISCUSSION

It is still unclear how *MLL*-PTD contributes to AML. A *Mll*^PTD/WT^ mice model was reported not to develop leukemia, but characterized by a proliferative advantage, abnormal self-renewal capability, and myeloid differentiation blockage in hematopoietic stem/progenitor cells [19]. Because *MLL*-PTD alone does not generate leukemia, acquisition of additional cooperating mutations is required for the development of AML. To understand the genetic background and determine whether the prognosis of *MLL*-PTD is influenced by any additional genetic aberration, we comprehensively investigated the pathobiologically important genetic aberrations known to be involved in myeloid neoplasms including DNA methylation, chromatin modifiers, activating signaling, myeloid transcription factors, and tumor suppressors in a large cohort of patients with *MLL-PTD* associated AML. We observed that *MLL*-PTD rarely occurred alone in AML. More than 90% of AML patients with *MLL-PTD* had at least one additional gene mutation. The most common concomitant gene mutations in *MLL*-PTD associated AML were *FLT3*-ITD, followed by *DNMT3A* mutation, *RUNX1* mutation, *IDH1/IDH2* mutation, and *TET2* mutation.

Of the class I mutation involving activated signaling pathways, *FLT3* mutations were the most common concomitant gene mutations in *MLL*-PTD associated AML, with a frequency of 45% for *FLT3*-ITD, and up to 58% if *FLT3*-TKD was included. Other class I mutations, such as *PTPN11*, *NRAS*, *KRAS*, and *CSF1R* mutations, were less frequently observed. Of the class II mutation involving hematopoietic transcription factors, we observed that *RUNX1* mutations were the most frequent co-existed gene mutation. Other class II mutations, such as double *CEBPA* and *NPM1* mutation, were absent in *MLL*-PTD associated AML. It has been reported that *RUNX1* mutations were significantly associated with *MLL*-PTD [20, 21]. The high frequency of *RUNX1* mutation and absent of *NPM1* or double *CEBPA* mutations might contribute in the poor outcome of patients with *MLL*-PTD AML. Gene mutations involving DNA methylation were common, with *DNMT3A*, *IDH1/IDH2* and *TET2* mutations accounting for 62% of patients with *MLL*-PTD associated AML. The high frequency of *FLT3*-ITD and mutations in epigenetic and/or chromatin remodeling-associated genes were generally in line with other cohorts with smaller patient numbers [10, 22, 23]. Another study including 225 *MLL*-PTD AML patients examined 12 genes published as an abstract, in which the frequency of *DNMT3A*, *FLT3*-ITD, *IDH1*, *IDH2*, and *RUNX1* mutations were 44.7%, 33.3%, 14.5%, 29.4%, and 25.8% [24]. However, gene mutations in *DNMT3A*, *NRAS*, *KRAS*, and *TP53* were only examined in half of their patients, and *TET2*, *EZH2*, *PTPN11*, and *CSF1R* mutations were not analyzed.

Taking the functional classification of genes into consideration, the most frequent concurrent gene mutations were genes of activating signaling pathways and epigenetic modifiers. The cooperative functions of *MLL*-PTD with *FLT3*-ITD mutations in the leukemogenesis of AML have been studied by Zorko et al, who demonstrated that the *Mll*^PTD/wt^:*Flt3*^ITD/wt^ double knock-in mice spontaneously developed AML and recapitulated the cytogenetic and molecular characteristics of human *MLL*-PTD and *FLT3*-ITD AML [25]. It is recently found that RUNX1 physically and functionally interacts with MLL, thereby regulating the epigenetic status of critical cis-regulatory elements for hematopoietic genes [26, 27]. In addition to the prevalent *FLT3*-ITD and *RUNX1* mutations in our study, *MLL*-PTD and gene mutations regulating DNA methylation frequently coexisted in our *MLLI*-PTD AML patients. As *MLL*-PTD is a regulator of histone modifications and frequently associated with other gene mutations involving DNA methylation, it is suggested that mechanistic interaction or crosstalk between MLL and other epigenetic regulators, especially DNMT3A, TET2 and IDH1/IDH2, may exist for the regulation of multiple hematopoietic related genes. However, the mechanism of interaction between MLL and DNMT3A, TET2 or IDH1/IDH2 was unclear, which significantly veiled the roles of epigenetic regulators in leukemogenesis, and restrict the development of new therapies. The underlying mechanism of aberrant DNA methylation or chromatin modulation in the leukemogenesis of *MLL*-PTD associated AML merits further investigation.

The present study is one of the largest series to specifically investigate the impact of additional genetic aberrations in *MLL*-PTD associated AML. We found that in addition to older age, *DNMT3A* mutations conferred an inferior OS in *MLL*-PTD AML patients. Multivariate analysis for age and *DNMT3A* mutation was not performed because the proportional hazard assumption was not met. Our findings suggest that the detection of *DNMT3A* mutations in *MLL*-PTD associated AML can therefore be recommended to identify the subgroup of patients with extremely poor outcome. In addition, the detection of gene mutations involving epigenetic regulators may also provide potential molecular targets or tailored treatment. Several promising treatment strategies were recently reported, including the *in vivo* benefit of targeting aberrant epigenetics in *MLL*-PTD associated AML patients [28], the anti-leukemic activity of liposomal bortezomib delivered as a single agent in the *Mll*^PTD/wt^:*Flt3*^ITD/wt^ double knock-in mice through the pharmacologic increase in *miR-29b* [29], and dose intensification of anthracycline in induction therapy to improve the survival of AML patients with *DNMT3A* mutations and/or *MLL*-rearrangement [30]. Very recently, Traina et al. reported that *DNMT3A* and/or *TET2* mutations were the independent predictors for a favorable response to DNMT inhibitors of 5-azacytidine and/or decitabine in myelodysplastic syndromes and related neoplasms [31]. Moreover, the preliminary data of an ongoing clinical trial with decitabine with or without bortezomib showed *DNMT3A* mutations conferred a better response to the hypomethylating agents in AML patients [32]. IDH is another promising target. Chaturvedi et al. recently reported that an inhibitor targeting mutant IDH1 induced apoptosis and decreased colony formation in methylcellulose of IDH1-mutant human primary bone marrow cells [33]. Wang F *et al.* also demonstrated that treatment with IDH2/R140Q inhibitor induced differentiation in TF-1 erythroleukemia and primary human AML cells *in vitro* [34]. *FLT3* mutations were common in patients with *MLL*-PTD AML. *FLT3* inhibitor to target FLT3 receptor tyrosine kinase is a potential treatment strategy which is under development or clinical trials [35–37]. In addition, *FLT3*-ITD can be monitored for minimal residual disease by using next generation sequencing to detect outgrowth of variable *FLT3*-ITD mutant clones during relapse in a single test [38, 39]. In the future, tailored, molecular directed combination therapy will hold promise to improve the outcome of patients with *MLL*-PTD AML who have high frequency of gene mutations in epigenetic regulators and activated signaling pathways.

In summary, we analyzed gene mutations in 98 patients with *de novo MLL-PTD* associated AML. We found that nearly two thirds of AML patients with *MLL-PTD* had at least one gene mutation involving in epigenetic regulators or activating signaling pathways. *DNMT3A*, *IDH1/IDH2* and *TET2* mutations were most common and *DNMT3A* mutation was associated with a poor outcome. We also confirmed that *FLT3*-ITD and *RUNX1* mutations frequently coexisted with *MLL*-PTD in AML patients. Our findings suggested that *MLL-PTD*, cooperated with other gene mutations involving epigenetic regulators, might result in deregulated gene expression and the leukemogenesis of AML. The cooperative biological function of *MLL*-PTD and other mutations in epigenetic regulators warrants further study. As AML patients with *MLL*-PTD even received intensive therapy such as stem cell transplantation still died of the disease, novel target therapy and tailored treatment according to their concomitant genetic aberrations will hold promise to improve the outcome of patients with *MLL*-PTD associated AML.

## MATERIALS AND METHODS

### Patient samples

Analyses of diagnostic bone marrow samples were carried out in 98 patients with *de novo* AML harboring *MLL*-PTD in Chang Gung Memorial Hospital and Mackay Memorial Hospital. G-banding method was used for karyotypic analysis. Cytogenetic categorization of favorable-, intermediate- and adverse-risk groups followed the criteria recommended by ELN guidelines [15]. Mutational analyses for *MLL*-PTD, *DNMT3A*, *IDH1* and *IDH2* were performed for all patients (*n* = 98), and *TET2*, *ASXL1* and *EZH2* were performed for 94 patients. Data of other molecular markers, including gene mutations in class I of activated signaling pathways (*FLT3*-ITD, *FLT3*-TKD, *JAK2*^V617F^, *NRAS, KRAS*, *CKIT*, *CSF1R*, *PTPN11*), class II of transcription pathways (*RUNX1*, *NPM1*, *CEBPA*), and tumor suppressor genes (*WT1*, *TP53*) were available for all patients. The study was approved by the Institutional Review Board of Chang Gung Memorial Hospital and Mackay Memorial Hospital.

### Detection of gene mutations

Cell fractionation, DNA or RNA extraction, and cDNA preparation were performed as previously described [16]. *MLL*-PTD was screened by reverse transcriptase (RT)-PCR and confirmed by real-time quantitative RT-PCR TaqMan assay using the primer sequences described in details in Supplementary Tables S1–S2. The 30 normal subjects had a normalized copy number (NCN) of 0.00008–0.0012 for e9e3 and 0.00029–0.00073 for e11e3, respectively. Sixty-nine patients had repetition of e9e3 with median NCN of 1.25 (range 0.21–9.78), and 29 patients had repetition of e11e3 with median NCN of 1.04 (range 0.44–5.78). The genomic DNA-PCR or reverse transcriptase-PCR assays for the detection of *FLT3*-ITD, *FLT3*-TKD, *CKIT* and *CSF1R* mutations, point mutations at codons 12, 13, and 61 in exons 1 and 2 of *NRAS* and *KRAS* genes, whole coding sequences (exons 3–8) of *RUNX1* mutations, mutated genes of *CEBPA*, *PTPN11*, *JAK2*^V617F^, *NPM1*, exons 4–10 of *TP53,* exons 1–3 and exons 7–9 of *WT1*, whole exons of *DNMT3A* and *TET2,* exon 4 of *IDH1* and *IDH2*, *DNMT3A*, and exon 12 of *ASXL1* were carried out as previously described [17]. For the detection of *EZH2* mutation, we used denaturing high performance liquid chromatography as the screening assay followed by direct sequencing for abnormal PCR products as described before [16].

### Statistical analysis

Fisher's exact test, the χ^2^ analysis, and Wilcoxon's rank-sum test were used whenever appropriate to make comparisons between groups. Estimates of survival were calculated according to the Kaplan-Meier method. Comparisons of estimated survival curves were analyzed by the log-rank test. In all analysis, the *P*-values were two-sided and considered statistically significant when values lower than 0.05. Statistical analysis was carried out by SPSS version 17.0 software (SPSS Inc, Chicago, IL).

## SUPPLEMENTARY FIGURES AND TABLES




