# Supplementary material for: High frequency of additional gene mutations in acute myeloid leukemia with MLL partial tandem duplication: DNMT3A mutation is associated with poor prognosis
Source: Oncotarget. 2015 Sep 8;6(32):33217–25. doi: 10.18632/oncotarget.5202 (PMC4741760; doi:10.18632/oncotarget.5202)
Supplement: Supplementary file 1 [file oncotarget-06-33217-s001.pdf]

SUPPLEMENTARY FIGURE AND TABLES

(A)

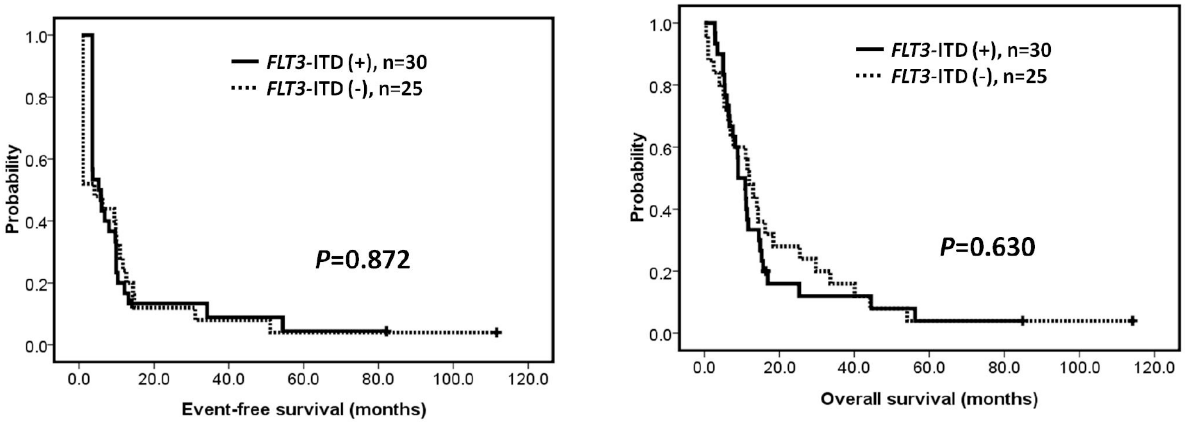

(B)

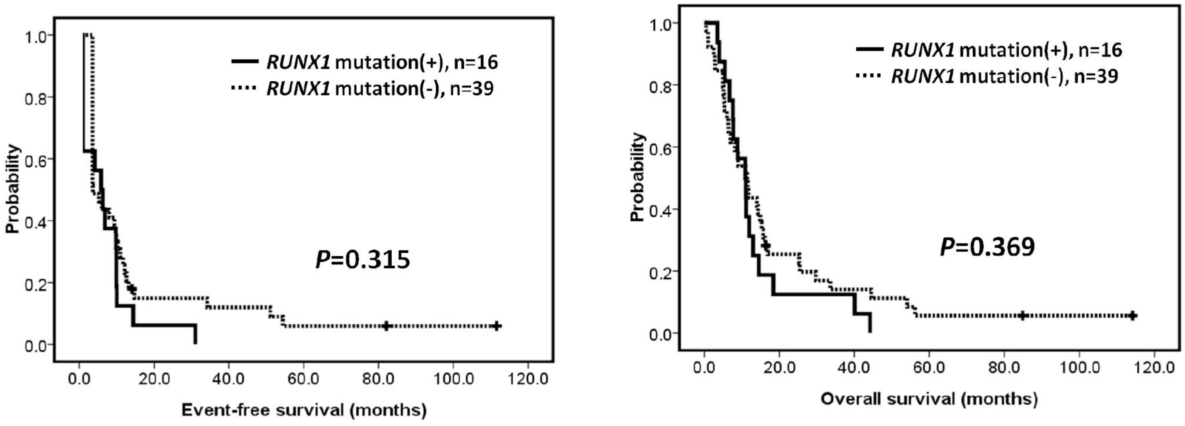

Supplementary Figure S1: Survivals of patients with *MLL*-PTD AML. Kaplan-Meier estimate of event-free survival (left panel) and overall survival (right panel) in *MLL*-PTD AML patients according to the mutation status of **A.** *FLT3*-ITD, **B.** *RUNX1*. (*Continued*)

(C)

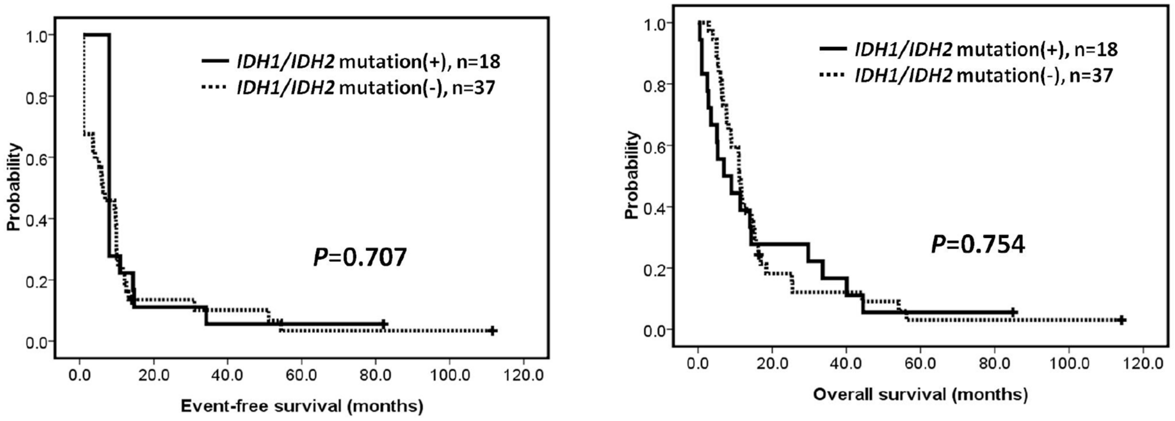

(D)

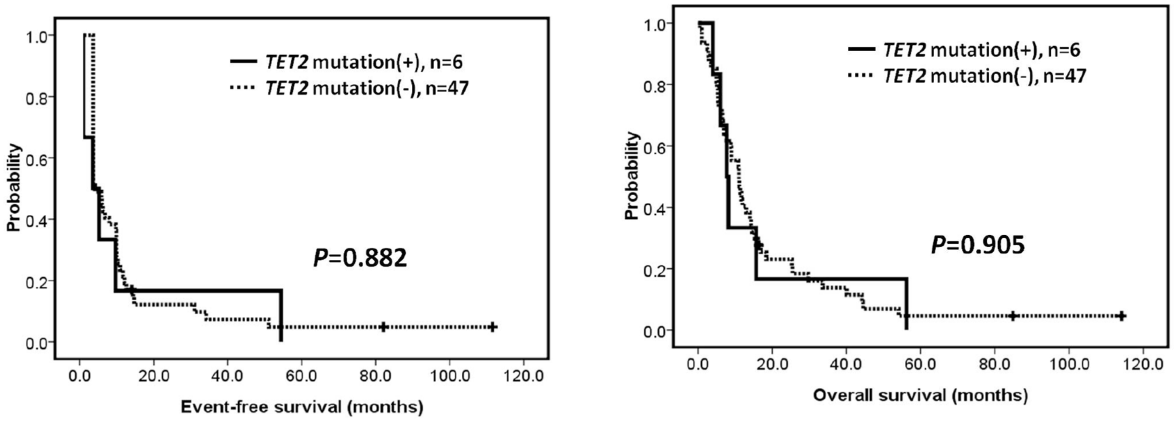

(E)

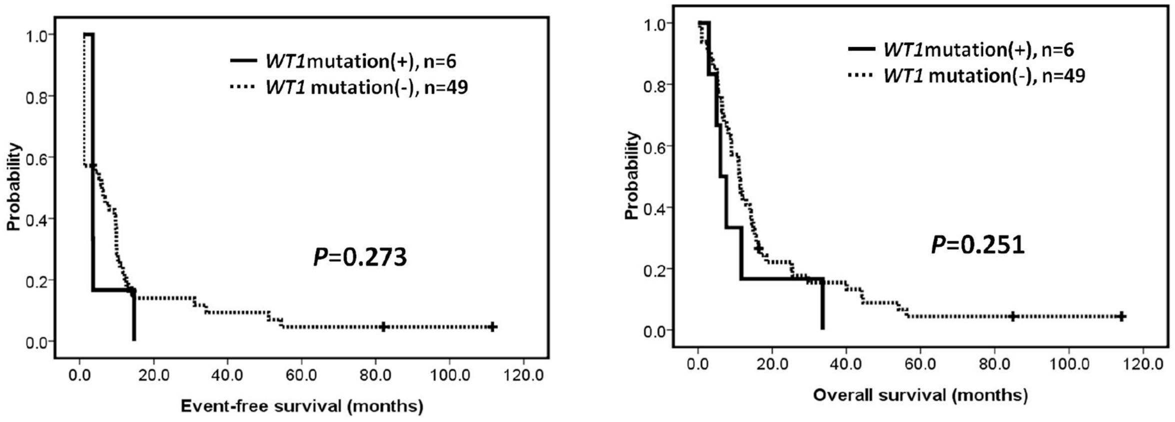

Supplementary Figure S1: (*Continued*) Survivals of patients with *MLL*-PTD AML. C. *IDH1/IDH2,* **D.** *TET2* and **E.** *WT1*.

Detection of *MLL*-PTD

Supplementary Table S1: Primer sequences for the screen of *MLL*-PTD and *ABL1* genes by RT-PCR

Size of PCR product	Primers	Primer sequences (5′ → 3′)
MLL-PTD PCR (655 bp)	MLLe (forward primer)	CCTGAATCCAAACAGGCCACCACT
	MLL3.1c (reverse primer)	AGGAGAGAGTTTACCTGCTC
PCR internal control (201 bp)	ABL-5F(forward primer)	TTCAGCGGCCAGTAGCATCTGACTT
	ABL-3R (reverse primer)	TGTGATTATAGCCTAAGACCCGGAG

MLL-PTD (e9e3) PCR product = 654 bp,
MLL-PTD (e11e3) PCR product = 900 bp
ABL1 PCR product = 201 bp

Supplementary Table S2: Primer sequences for the detection of *MLL*-PTD and *ABL1* genes by real-time quantitative RT-PCR TaqMan assay

Primer/probe	position (size)	Sequence (5′ → 3′)
MLL-ex9-F	4036–4058 (23)	GTCCAGAGCAGAGCAAACAGAAA
MLL-ex11-F	4332–4351 (20)	TGTGCCAGTAGTGGGCATGT
MLL-ex2(3)-R	572–560 (23)	AATTTCGGTCAGAGCCACTTCTA
MLL-PTD probe	494–520 (27)	CAGAGTGCGAAGTCCCACAAGGTCTCC
ABL-QF	589–617(29)	TGGAGATAACACTCTAAGCATAACTAAAGGT
ABL-QR	691–712 (21)	GATGTAGTTGCTTGGGACCCA
ABL probe	567–594 (28)	CCATTTTTGGTTTGGGCTTCACACCATT

Positions according to accession numbers D14540 (*MLL*) and M14752 (*ABL1*).
Reference values (NCN = *MLL*-PTD/*ABL1*)
MLL-PTD(e9e3) Normal range (N = 30): 0.00008~0.0012
MLL-PTD(e11e3) Normal range (N = 30): 0.00029~0.00073
The normal upper limit of NCN for e9e3 *MLL*-PTD is <0.0012
The normal upper limit of NCN for e11e3 *MLL*-PTD is <0.00073

Supplementary Table S3: Patient treatment and outcome according to age groups

Age groups	<18 years	18–59 years	>=60 years
**Treatment**			
Standard 3 + 7 chemotherapy	1	41	9
TPOG 2002 AML protocol	4	0	0
Low dose chemotherapy	0	0	2
Supportive care only	0	6	35
**Complete remission rate**	100% (5/5)	48.8% (20/41)	77.8% (7/9)
**Allogeneic stem cell transplantation**	1	7	0
**Median OS (months)**[+]	16.9 (5.3–28.5)	8.2 (3.5–12.8)	2.8 (0.2–5.4)
**Median EFS (months)**[+]	10.3 (8.4–12.2)	0	0

[+] **Values: Median with 95% confidence interval**.

Supplementary Table S4: Clinical characteristics of *MLL*-PTD AML patients with *DNMT3A, TET2, IDH1/IDH2, FLT3*-ITD and *RUNX1* mutations.
