# Supplementary material for: High frequency of additional gene mutations in acute myeloid leukemia with MLL partial tandem duplication: DNMT3A mutation is associated with poor prognosis
Source: Oncotarget. 2015 Sep 8;6(32):33217–25. doi: 10.18632/oncotarget.5202 (PMC4741760; doi:10.18632/oncotarget.5202)
Supplement: Supplementary file 2 [file oncotarget-06-33217-s002.pdf]

Table S4. Clinical characteristics of *MLL*-PTD AML patients with *DNMT3A*, *TET2*, *IDH1/IDH2*, *FLT3*-ITD and *RUNX1* mutations

Features	***DNMT3A* mutation**		***P***	***TET2* mutation**		***P***	***IDH1/IDH2* mutation**		***P***	***FLT3*-ITD**		***P***	***RUNX1* mutation**		***P***
	**Positive (N=32)**	**Negative (N=66)**		**Positive (N=18)**	**Negative (N=76)**		**Positive (N=28)**	**Negative (N=70)**		**Positive (N=44)**	**Negative (N=55)**		**Positive (N=23)**	**Negative (N=76)**	
**Median age (years)**	61(20-84)	54(6-85)	0.053	71(34-84)	54(6-85)	0.004	54(6-86)	61(9-85)	0.351	54(9-85)	61(6-84)	0.246	51(6-80)	62(11-85)	0.202
**Male/female**	12/20	37/29	**0.085**	10/8	37/39	0.600	15/13	34/36	0.412	18/26	31/23	0.104	10/13	39/36	0.475
**Median Hemoglobin level (g/dl)**	7.8 (3.3-13.7)	7.2(3.6-11.6)	0.611	7.2(3.3-13.7)	7.4(3.6-13.7)	0.829	7.7(5.2-11.4)	7.2(3.3-13.7)	0.037	7.2(3.3-13.7)	7.7(3.8-11.4)	0.165	7.1(3.8-11.6)	7.5(3.3-13.7)	0.234
**Median platelet count (x10^9/l)**	41.0(3-900)	52.0(1-471)	0.705	48.0(12-303)	46.0(1-900)	0.686	31.0(3-900)	46.5(1-327)	0.662	49.5(1-327)	45.0(3-900)	0.645	48.0(1-327)	45.5(3-900)	0873
**Median WBC count (x10^9/l)**	45.4(1.6-451.4)	23.0(0.8-205.2)	0.135	51.8(5.1-255.0)	27.3(0.8-451.4)	0.136	11.3(0.8-451.4)	41.5(1-353.9)	0.059	63.5(1-451.4)	14.9(0.8-187.2)	0.001	59.2(1-203.7)	28.9(0.8-451.4)	0.123
**Median circulating blast (%)**	63.5(4.7-96.5)	64.0(0-98.3)	0.611	64.6(6.5-95.8)	62.9(0-98.3)	0.676	73.6(0-98.3)	63.3(0-95.8)	0.712	71.8(0-98.3)	61.7(0-94.0)	0.036	65.6(0-94.0)	60.1(0-98.3)	0.465
**Median marrow blast (%)**	82.1(36.4-99.4)	77.8(2.5-97.0)	0.256	77.8(45.2-99.4)	80.3(2.5-98.8)	0.851	77.8(42.7-96.2)	80.5(34.7-99.4)	0.096	82.9(2.5-99.4)	69.3(34.7-98.8)	0.001	85.3(36.4-99.3)	77.8(2.5-99.4)	0.130
**ELN cytogenetic risk group**			0.547			0.307			0.496			1.000			1.000
**Intermediate 1**	21	35		8	46		18	36		28	28		14	42	
**Intermediate 2**	6	13		5	14		3	16		10	9		5	14	
**Adverse**	0	1		0	1		0	1		0	1		0	1	
**FAB classification**						0.873			0.496			0.144			0.313
**M0/M1/M2**	0/10/14	5/14/29		0/5/8	5/18/32		3/7/14	2/17/29		1/15/16	4/9/27		2/7/8	3/17/35	
**M4/M5**	5/2	12/3		4/1	13/4		2/0	15/5		9/3	8/2		3/3	14/2	
**M6/M7**	0/1	3/0		0/0	3/1		1/1	2/0		0/0	3/1		0/0	3/1	
